# Mobile Application's Effect on Patient Satisfaction and Compliance in Total Joint Arthroplasty: A Systematic Review and Meta-analysis

**DOI:** 10.5435/JAAOSGlobal-D-22-00200

**Published:** 2023-09-06

**Authors:** Rubén Monárrez, Amin Mohamadi, Jacob M. Drew, Ayesha Abdeen

**Affiliations:** From the Department of Orthopaedic Surgery (Dr. Mohamadi, Dr. Drew), Beth Israel Deaconess Medical Center, Harvard Medical School, Boston, MA (Dr. Monárrez, Dr. Mohamadi, Dr. Drew, and Dr. Abdeen); the Sinai Hospital of Baltimore, Rubin Institute for Advanced Orthopedics, Baltimore, MD (Monárrez); and the Department of Orthopaedic Surgery, Boston Medical Center, Boston, MA (Abdeen).

## Abstract

Use of mobile applications to improve patient engagement is particularly promising in total joint arthroplasty (TJA) whereby successful outcomes are predicated by patient engagement. In accordance with published guidelines by the Preferred Reporting Items for Systematic Reviews and Meta-Analyses, studies were searched, screened, and appraised for quality on various search engines. Hedges' g or odds ratios of patient adherence were reported. Twelve studies met the inclusion criteria, and the average age of 9,521 patients included was 60 years. Six studies concluded that mobile applications improved patients' satisfaction, with Hedges' g revealing an effect size of 1.64 (95% confidence interval [CI] 0.90 to 2.37), *P* < 0.001, in favor of mobile applications increasing patient satisfaction. Six studies reported improvements in compliance demonstrating an odds ratio for improved adherence of 4.57 (95% CI, 1.66 to 12.62), *P* < 0.001. Two studies reported a reduction in unscheduled office or emergency department visits. With evolving reimbursement policies linked to outcomes paired with the exponentially increasing volume of TJA performed, innovative ways to efficiently deliver high-quality care are in demand. Our systematic review is limited by a dearth of research on the nascent technology, but the available data suggest that mobile applications may enhance patient satisfaction, improve compliance, and reduce unscheduled visits after TJA.

Smartphones have become ubiquitous devices. A recent Pew Research Center survey demonstrated that 81% of Americans own smartphones, including 79% of adults aged 50 to 64 years and 53% of those older than 65 years who owned a smartphone.^1^ Subsequently, a wide range of mobile applications have been developed to measure and improve health-related outcomes and patient satisfaction. Mobile applications are currently being used in a wide range of medical fields for patient engagement, monitoring, and education. Initially, these platforms were designed for the management of chronic illnesses (such as diabetes, hypertension, cardiovascular disease, and obesity) in a variety of groups and settings (pediatric and geriatric populations, minority communities, and low-resource regions).^2–4^ Most interventions have focused on sustained behavioral or lifestyle changes, such as medication adherence, increased physical activity, and smoking cessation; however, the sustainability of patient engagement through mobile applications diminishes over time as users develop “app-fatigue.”^5^ Mobile applications may have more of an effect when used for health states limited to a finite episode of care such as pregnancy and elective procedures.^6,7^

The field of gastroenterology pioneered the periprocedural use of mobile applications, specifically for patient engagement, and education for bowel preparation before colonoscopy with demonstrated improvements in patient satisfaction, quality of bowel preparation, and procedural outcomes.^8–10^ Mobile applications have since been adapted for perioperative use in a wide range of surgical fields (ophthalmology, colorectal surgery, urology, and orthopaedic surgery) to achieve a broad spectrum of goals pertaining to patient education, compliance with perioperative protocols, monitoring for postoperative complications, and collection of patient-reported outcomes and patient satisfaction.^11–18^

Within orthopaedic surgery, mobile applications are used for patient engagement for a variety of procedures, including lumbar diskectomy, anterior cruciate ligament (ACL) reconstruction, and total joint arthroplasty (TJA)^15–18^ (Figure 1). The utility of mobile applications is particularly promising in the field of TJA of the hip and knee in which the volume of cases is predicted to exponentially increase by 174% and 673%, respectively, by 2030.^19^ Patient engagement is critical for those undergoing TJA of the hip and knee to improve patient-physician relationship and dynamic interaction to optimize modifiable risk factors.

**Figure 1:**
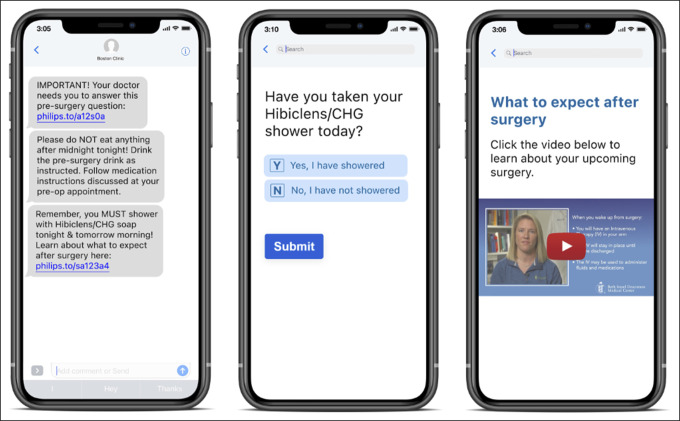
Screenshots showing an example of an internal mobile application developed to engage arthroplasty patients.

To our knowledge, there is no existing systematic review of the literature that evaluates the use of mobile applications used specifically for patients undergoing TJA of the hip and knee. Therefore, we conducted a systematic review and meta-analysis to determine the following: (1) Do mobile applications improve patient satisfaction? (2) Do mobile applications improve validated patient-reported outcomes? (3) Do mobile applications improve patient compliance with perioperative protocols? (4) Do mobile applications reduce the incidence of unscheduled postoperative visits in those undergoing TJA of the hip and knee? We hypothesized that mobile applications would improve overall patient perioperative care on all four of these points.

## Methods

### Search Strategy and Selection Criteria

In accordance with published guidelines by the Preferred Reporting Items for Systematic Reviews and Meta-Analyses, EMBASE, PubMed, medical literature analysis and register of systematic reviews (MEDLINE), and Web of Science were searched on July 2019.^20^ This review was registered before data collection with the international prospective register of systematic reviews (PROSPERO) international prospective register of systematic reviews (CRD42020144633). No funding was provided for this research. The search was conducted without language restrictions using various relevant keywords to the subject (see full search strategy attached as Appendix 1, http://links.lww.com/JG9/A299). A manual search of the gray literature was also conducted. The electronic search was updated in December 2021 to include recent publications.

### Inclusion and Exclusion Criteria

Screening for inclusion was conducted by two independent investigators (R.M. and A.A.). A third investigator (A.M.) resolved any conflicting assignments during the screening process. Inclusion criteria were all studies that evaluated the use of a mobile phone application on patient engagement, satisfaction, and outcomes after total joint replacement (TJR) of the hip and/or knee. Studies involving wearable devices were excluded. We excluded meeting presentations, abstracts, project proposals, and ongoing studies without data.

### Study Methodological Quality

All included studies were assessed for risk of bias: Two investigators (R.M. and A.A.) independently appraised the quality of the included studies using the Newcastle-Ottawa Scale 2^21^ and disagreements were resolved by a third reviewer (A.M.). To provide additional resolution of the quality of each study, an Agency for Healthcare Research and Quality standard score was also obtained and reported as poor, fair, or good in quality.^21^

### Primary and Secondary Outcomes

Two independent reviewers (R.M. and A.A.) extracted data according to a standardized template that included study design, number of included patients, intervention purpose, control groups used, main outcomes, and secondary outcomes. A third reviewer (A.M.) verified the accuracy of the collected data. When needed, we obtained raw data from authors who responded to data requests.^22,23^

### Search Results

**Figure 2:**
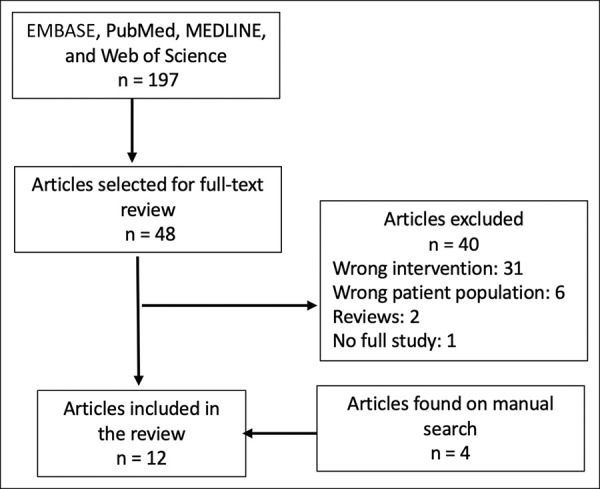
PRISMA flow diagram. Of the 12 studies that the met inclusion criteria for qualitative analysis, six studies also provided sufficient data to be included in the quantitative meta-analysis. PRISMA = Preferred Reporting Items for Systematic Reviews and Meta-Analyses

After the removal of duplicates, 197 studies were identified, of which 48 were selected for full review based on their abstract. We excluded 40 articles after reviewing the full text of the eligible studies and added two studies found in the gray literature search (Figure 2).

Twelve studies met the inclusion criteria for the qualitative analysis (Table 1). The average age of 9,521 patients included was 60 years. The average Newcastle-Ottawa Scale score was 7.5 of 9 possible points. Four^24–27^ of the 12 studies were given an Agency for Healthcare Research and Quality poor rating because they lacked control groups (these studies were not used in any of the quantitative analysis), and one^28^ was rated as fair because it failed to provide demographic comparisons between the cohorts (Appendix 2, http://links.lww.com/JG9/A300). These 12 studies encompassed a variety of orthopaedic practice settings including numerous large academic practices, 4 hospitals outside of the United States,^25,27,29,30^ and 3 studies focused on community practices in the United States.^23,26,31^ Various mobile applications were used throughout, including StreaMD, PreHab, iGetBetter, HealthLoop, preopPTEd, and self-developed applications.

**Table 1:** Study Characteristics

Reference	n (Intervention/Control)	Type of Study	Age (Mean Years)	Function of Mobile Intervention	Patient Satisfaction	Compliance With Perioperative Protocols	Reduction in Unscheduled Visits	AHRQ, NOS
Blocker et al, 2017^27^	11/NA	Observational	70.0	Collecting outcomes, post-op	High, positive opinions on interview	Not studied	Not studied	Poor, 6
Campbell et al, 2019^34^	76/83	RCT	60.2	Adherence and monitor narcotic use, post-op	Higher, felt care team motivated them through their recovery (relative risk (RR), 2.5]; *P* < 0.001) and more encouraged by the care team ([RR, 2.2]; *P* < 0.001).	Improved, more likely to partake in rehab exercises ([RR = 1.7], *P* < 0.001) and improved 3 wk ROM	Improved, stopped narcotics 10 days sooner (*P* < 0.001) and fewer ED and office visits (*P* < 0.001)	Good, 9
Chugtai et al, 2019^31^	114/362	Prospective	65.1	Improve participation in prehab, pre-op	Not studied	Improved, as reported by shorter LOS and higher likelihood of favorable discharge disposition (*P* < 0.001)	Not studied	Good, 8
Day et al, 2019^22^	30/26	Cohort	59.2	Reminders, supportive, and complication monitoring, pre-op and post-op	Higher, more likely to feel the care team included them in their decisions (*P* = 0.024)	Moderate, 63% actively used the mobile application	Not studied	Good, 9
Garnier et al, 2018^29^	298/301	Prospective	49.0	Adherence through a mobile reminder, pre-op	Higher, intervention group more satisfied with the method of reminder (87.2% vs. 63.8%, *P* < 0.001)	Improved, as measured by preoperative compliance ([OR = 1.9], *P* < 0.0001)	Not studied	Good, 9
Gwam et al, 2019^23^	1111/1030	Retrospective	63.6	Adhere to protocols and monitor complications, pre-op and post-op	Higher, as demonstrated by patient satisfaction scores and satisfaction with communication with doctors (*P* < 0.001)	Not studied	Improved, decrease in 90-day complications (*P* = 0.035) and decrease in severe pain requiring unscheduled visit (*P* = 0.026)	Good, 9
Kim et al, 2016^24^	13/NA	Observational	68.8	Adhere to pre-op meds and PT and collect outcomes, pre-op and post-op	Not measured	Moderate, 59% adherence to PT exercises	Not studied	Poor, 6
Park et al, 2017^30^	19/21	RCT	66.0	Monitoring function and collecting outcomes, post-op	No difference in satisfaction	Not studied	Not studied	Good, 8
Premkumar et al, 2018^28^	183/5,277	Prospective	59.4	Adherence and complication monitoring, post-op	Not studied	Improved, response rate (96.1% vs 66.6%, *P* < 0.001)	Not studied	Fair, 8
Rosner et al, 2018^26^	371/NA	Observational	56.5	Adherence and complication monitoring, post-op	Not studied	High, 76.8% survey completion rate	Accurate, self-reported hospital admission (k = 0.80, agreement of 0.99) and pulmonary embolism (PE) (k = 1, agreement of 1)	Poor, 6
Scheper et al, 2019^25^	69/NA	Observational	68	Adherence and monitoring for complications, post-op	High, 8.2/10 on a Likert scale	Yes, 59.4% used the application until the end of the study	Accurate, 80% concordance with physician-reported outcomes	Poor, 4
Soeter et al, 2018^33^	63/63	RCT	61.5	Improve PT participation, pre-op and post-op	No difference in the western ontario and mcmaster universities arthritis index (WOMAC) scores	Improved, as reported by meeting PT goals sooner (*P* < 0.001)	Not studied	Good, 8

AHRQ = Agency for Healthcare Research and Quality rating, NOS = Newcastle-Ottawa Scale score, PT = physical therapy, RCT = randomized controlled trial

### Quantitative and Qualitative Analysis

In the meta-analysis, the satisfaction scores, SD, and number of participants were used to calculate Hedges' g and adjusted standardized mean difference when comparing the continuous outcomes measured with a variety of tools for each study. Hedges’ g indicates the size of the intervention effect in each study relative to the variability observed among studies by difference of means in study groups divided by the pooled SD. If data were presented in 95% confidence intervals (CIs), the SD is calculated with the following formula: SD = 95% CI/1.96 × √n. The standardized mean difference is considered to represent trivial (<0.20), small (0.20 to 0.49), medium (0.50 to 0.79), or large (>0.80).

To compare patient adherence with perioperative protocols across studies, we used the number of compliant and noncompliant patients in each intervention or control group to calculate pooled odds ratio. Perioperative protocols ranged from adherence with physical therapy (PT), logging outcomes such as opiate consumption, and satisfaction throughout the surgical journey.

Heterogeneity among studies was assessed using Cochran's Q and I^2^ statistics and analyzed further if significant (*P* < 0.1). Where heterogeneity still existed (*P* < 0.1) and could not be further explained, we used a random-effects meta-analysis. A funnel plot was drawn and inspected visually, and the Begg-Mazumdar test was used to evaluate for the presence of publication bias.^32^ Statistical analysis was done using Comprehensive Meta-Analysis version 2 (Biostat) software.

## Results

### Patients' Satisfaction

Eight studies reported on the effect of mobile applications on patient satisfaction. Six (75%) concluded that mobile applications improved patient satisfaction after TJA surgery, and two (25%) reported no differences when compared with routine care. There was a wide range of methods of reporting on patients' satisfaction, including surveys and structured interviews.^23,25,27,29–31,33,34^ Survey questions capturing patients' satisfaction included direct satisfaction questions on Likert scales and improvements in their perception and communication with their care team.

**Figure 3:**
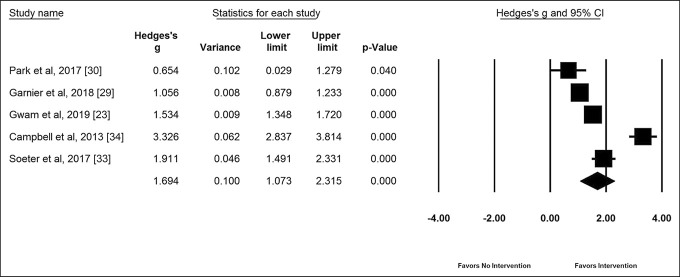
Forest plot showing pooled effect size of mobile applications on patient satisfaction. A larger Hedges' g value indicates increased patient satisfaction with mobile applications when compared with standard of care.

Five studies^23,29,30,33,34^ containing 1,450 patients were pooled, which demonstrated that mobile applications improved patient satisfaction (Figure 3). The sample sizes of the included studies ranged from 40 to 2,141 patients (mean = 613; interquartile range [IQR] = 126 to 599). There was a large heterogeneity among the studies (I^2^ = 95.40%; *P* < 0.001); therefore, we used random-effects meta-analysis. The overall size of the effect was 1.7 (95% CI, 1.07 to 2.3), *P* < 0.001, indicating a large size of effect in favor of medical mobile applications to increase patient satisfaction. A funnel plot (Appendix 3A, http://links.lww.com/JG9/A301) and the Begg-Mazumdar test indicated no significant publication bias among these studies (*P* = 0.4).

### Validated Patient-Reported Outcomes

Only a single study^33^ used a validated functional outcome score (the Western Ontario and McMaster Universities Arthritis Index). Because of this, there were insufficient data to conduct a quantitative or qualitative analysis.

### Total Joint Arthroplasty Patient Compliance With Perioperative Protocols

Nine studies reported on increasing compliance with perioperative protocols. Six of them (67%) reported large improvements in compliance, and three (33%) had moderate improvements when compared with standard of care.^22,24–26,28,29,31,33,34^ Five reported compliance with PT exercises, and four reported on survey completion (eg, outcomes and opiate use).

**Figure 4:**
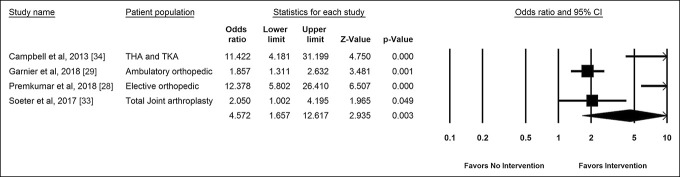
Forest plot showing pooled odd ratios of mobile applications effect on patient adherence with perioperative protocols. A higher odds ratio indicates greater odds of adhering to the perioperative protocol with mobile applications when compared with standard of care.

Four studies^28,29,33,34^ containing 6,341 patients reported sufficient quantitative data for meta-analysis on patient adherence, with perioperative protocols using mobile applications when compared with standard of care (Figure 4). The sample sizes of the included studies ranged from 126 to 5,460 patients (mean = 1,586; IQR = 150 to 1,814). There was a large heterogeneity among the studies (I^2^ = 89%; *P* < 0.001); therefore, we used random-effects meta-analysis. The pooled odds ratio effect size was 4.6 (95% CI, 1.7 to 12.6), *P* < 0.001, suggesting that mobile applications do increase patient adherence with perioperative protocols. A funnel plot (Appendix 3B, http://links.lww.com/JG9/A302) and the Begg-Mazumdar test indicated no significant publication bias among these studies (*P* = 0.3).

### Prevention of Unscheduled Visits

Four articles reported on mobile applications ability to reduce postoperative unscheduled visits, and all four supported their use.^23,26,33,34^ Two articles reported on the reduction of unscheduled office or emergency department visits when compared with control groups, and two reported on the accuracy between patient self-reported postoperative monitoring through mobile applications and provider findings. Owing to the limited quantitative data available, there were insufficient data to conduct a pooled quantitative analysis of this outcome.

## Discussion

In the current climate of evolving reimbursement policies that are increasingly linked to surgical outcomes paired with the exponentially increasing volume of TJA performed in the United States, innovative ways to efficiently deliver high-quality care at low cost are in demand.^19,35,36^ Mobile applications may be one method to monitor and improve outcomes in the perioperative period after TJA of the hip and knee; however, there remains a dearth of research on the nascent technology.

Limitations of this study include the paucity of research available. Further limiting our available data was the lack of control groups in some studies, which prevented their inclusion in our meta-analysis. Future research is need with robust control groups, such as sham platforms, to further delineate the effect size of mobile applications on patient satisfaction and compliance.^37^

A second limitation of our review was the heterogeneity of interventions and outcome collection across studies. Only a single study reported our primary outcome, satisfaction, with validated patient-reported outcomes, while all other studies used various unvalidated tools, adding to the limitations of using satisfaction as an outcome.^38^

Finally, there were several domains of bias in our review. Available studies were skewed toward large urban academic hospitals. In addition, three of the studies had one or more authors reporting conflict of interests directly related to the mobile applications they were studying. Future research is needed to evaluate mobile applications generalizability to community and rural settings.

A perceived limitation of mobile application use in TJA is that the technology may be less accessible to most patients undergoing hip and knee arthroplasty. Previous studies have identified a high interest in TJA patients using mobile applications, and comparable patient populations have reported a willingness to use technological interventions if age-related barriers (dexterity and vision) could be mitigated.^39,40^ The average age of patients in this systematic review was 60 years, which is indeed representative of the age of the patients typically undergoing TJR. The data reveal the enthusiasm and ability to engage technological interventions of this population. Our findings indicate high rates of participation and numbers agreeing to participate again in mobile applications in future surgeries.^22^ Furthermore, no difference was observed between TJA patients younger or older than 65 years in their engagement with the mobile application intervention.^26^ Future generations of TJA patients will likely be even more accustomed to mobile phones; thus, enthusiasm and comfort to engage in such interventions are expected to increase.

Communication through a mobile application provides the surgeon and care team an opportunity to engage patients in a mutually convenient manner outside of face-to-face episodes of care. The use of mobile application platforms may improve patient perception of the quality of communication with their physicians (particularly in comparison with the use of conventional telephone calls) and enhances satisfaction with their care.^23,29,41,42^ In addition, patients who participated in mobile intervention groups in TJA in this review reported a greater sense of shared decision making on their Press Ganey/HCAHPS scores (*P* = 0.024)^22^ and felt more encouraged by the care team ([RR, 2.2]; *P* < 0.001).^34^

Patient compliance is a challenge in all aspects of medicine. Previous reports have demonstrated that 78% of TJA patients are noncompliant with preoperative disinfection protocols and compliance is not appreciably improved with preoperative educational classes among them.^43,44^ Patients in this systematic review were more likely to be compliant with preoperative protocols and prepared for their surgery in comparison with those who received a phone call. In addition, although it was outside the scope of this review to assess the utility of preoperative PT (“prehabilitation”) in TJA, our systematic review identified increased compliance of TJA prehabilitation among those participating in a mobile application intervention, which was often correlated with improved outcomes.^24,31,33,34,45–48^ Overall, there seemed to be no decline in patient-observed engagement over time, which may be due to the relatively short and finite episode of care during which the applications were used.^24^

Another suggested benefit of mobile applications is the ability to monitor patients outside of the hospital. Our systematic review reveals mobile applications use to be associated with a decrease in episodic and unplanned healthcare visits. Furthermore, clinical findings acquired by mobile applications were consistent with those of in-person visits.^23,25,26,34^ In addition, this study revealed an overall increased rate of postoperative survey completion with the use of a mobile application in comparison with in-office forms.^26–28,49^ The use of a mobile application may be an opportunity for surgeons to effectively gather valuable data regarding patient feedback and functional outcomes and may help reduce unscheduled visits because of the added and improved communication such an intervention provides.

## Conclusion

The findings of this systematic review and meta-analysis suggest that mobile applications may enhance patient satisfaction, improve compliance with perioperative protocols, and improve patient engagement throughout TJA in the practices in which they were tested. However, the existing literature is insufficient and inadequate to determine the widespread usability of mobile applications in this population. Bias was pervasive in the extant of the studies, and a large portion of the studies (25%) had conflicts of interest.

Additional investigation is needed to validate the utility of mobile applications in patients undergoing TJA and to investigate their impact metrics that influence hospital and surgeon reimbursement, including postoperative complications, inpatient length of stay, and readmission rates. With additional refinements, mobile applications could be adapted to a variety of cultures and languages to improve the experience of minority groups at risk of receiving disproportionate quality of care.^50^ Additional studies are needed to evaluate cost-effectiveness as well as feasibility and accessibility of mobile application use inclusive of all patient populations and practice settings.
